# Expanding the phenotypic and genetic spectrum of *GTPBP3* deficiency: findings from nine Chinese pedigrees

**DOI:** 10.1186/s13023-024-03469-3

**Published:** 2024-12-24

**Authors:** Yaojun Xie, Keyi Li, Li Yang, Xiaofei Zeng, Zhehui Chen, Xue Ma, Luyi Zhang, Yuwei Zhou, Liqin Jin, Yanling Yang, Xiaoting Lou

**Affiliations:** 1 Laboratory Medicine Center, Department of Genetic and Genomic Medicine, Zhejiang Provincial People’s Hospital, Affiliated People’s Hospital, Hangzhou Medical College, Hangzhou, Zhejiang China; 2 Genetics Center of Obstetrics and Gynecology, Obstetrics and Gynecology Hospital of Fudan University, Shanghai, China; 3 Key Laboratory of Laboratory Medicine, Ministry of Education, Zhejiang Provincial Key Laboratory of Medical Genetics, School of Laboratory Medicine and Life Sciences, Wenzhou Medical University, Wenzhou, Zhejiang China; 4 Department of Pediatrics, Clinical Research Center for Children Neurodevelopmental Disabilities of Hunan Province, Xiangya Hospital, Central South University, Changsha, Hunan China; 5 Department of Pediatrics, Peking University First Hospital, Beijing, China; 6 Department of Scientific Research, Zhejiang Provincial People’s Hospital, Affiliated People’s Hospital, Hangzhou Medical College, Hangzhou, Zhejiang China

**Keywords:** Mitochondrial diseases, Oxidative phosphorylation, *GTPBP3*, Genetic hotspot, τm^5^(s^2^)U modification

## Abstract

**Background:**

GTPBP3 catalyzes τm^5^(s^2^) U biosynthesis at the 34th wobble position of mitochondrial tRNAs, the hypomodification of τm^5^U leads to mitochondrial disease. While twenty-three variants of *GTPBP3* have been reported worldwide, the genetic landscape in China remains uncertain.

**Methods:**

By using whole-exome sequencing, the candidate individuals carrying *GTPBP3* variants were screened and identified. Pathogenicity analysis of variants was biochemically verified by patients-derived immortalized lymphocytes and cell models.

**Results:**

Through whole-exome sequencing, thirteen variants associated with *GTPBP3* were identified in nine Chinese pedigrees, with eight of these variants being newly reported. Affected individuals displayed classic neurologic phenotypes and heart complications including developmental delay, seizures, hypotonia, exercise intolerance, and hypertrophic cardiomyopathy. Additionally, they displayed new symptoms such as eye problems like strabismus and heart issues related to valve function. Studies conducted on patient-derived cells provided evidence of reduced levels of GTPBP3 and impairment in mitochondrial energetic biogenesis. Re-expressing *GTPBP3* variants in knockout cell lines further defined the pathogenicity of the novel variants. Analysis of the genetic spectrum in the Chinese population highlighted a concentration in exons 4 and 6, with c.689A > C being the prominent hotspot.

**Conclusion:**

Our findings emphasize the extensive clinical and genetic implications of *GTPBP3*-related mitochondrial disorders, particularly within the Chinese population, but further investigations are needed to explore the phenotype-genotype correlation.

**Supplementary Information:**

The online version contains supplementary material available at 10.1186/s13023-024-03469-3.

## Introduction

Mitochondria contain over 1500 proteins, with the majority being encoded by the nuclear genome. The mitochondrial genome encodes 13 subunits of the mitochondrial oxidative phosphorylation (OXPHOS) system and is further transcribed and translated into proteins by the mitochondria’s internal system [1, 2]. Initially, mitochondrial DNA (mtDNA) transcriptions produce mitochondrial ribosomal RNAs (mt-rRNAs) and mitochondrial transfer RNAs (mt-tRNAs) required for translation. According to the mt-mRNA template, mature mt-tRNAs are enzymatically catalyzed by aminoacyl tRNA synthase to transport amino acid to the mitochondrial ribosome, facilitating the synthesis of new polypeptide chains [3]. The maturation of mt-tRNA involves endonuclease cleavage, 3 'end addition of CCA, and nucleobase modification. Post-transcriptional nucleobase modification plays a crucial role in preserving the stability and spatial conformation of mt-tRNA molecules, ensuring efficient and accurate decoding [4–6]. Presently, 18 types of nucleobase modifications have been identified on 22 different mt-tRNAs. Distinct modifications at various locations may correspond to diverse physiological outcomes [3].

GTPBP3 is a highly conserved mt-tRNA modifying enzyme that plays a crucial role in the biosynthesis of τm^5^ (s^2^) U, which modifies the 34th nucleobase of mt-tRNALeu^(UUR)^, mt-tRNA^Trp^, mt-tRNA^Glu^, mt-tRNA^Gln^ and mt-tRNA^Lys^ [7 –9]. This modified nucleobase, also referred to as "wobble base", is essential for limiting the range of wobble base pairs, which helps to maintain an efficient decoding rate [10]. Defects in *GTPBP3* often result in combined oxidative phosphorylation deficiency 23 (COXPD23), with patients clinically manifesting a series of symptoms, such as hypotonia, seizures, dyspnea, feeding difficulties, developmental retardation, fatigue, and limited vision. The examination results often suggest myocardial hypertrophy, lactic acidosis, and T2 hypersignal in the bilateral thalamus, basal ganglia, and brain stem [11–13]. Currently, only 21 cases of *GTPBP3* deficiency have been reported [11–17]. Despite the collection of over 300 variants of ClinVar (www.ncbi.nlm.nih.gov/clinvar), the pathogenicity of the majority of these remains unknown, and there is a lack of clinical information to facilitate accurate clinical diagnosis. Consequently, there is a need to enhance our understanding of the clinical spectrum and genetic spectrum associated with *GTPBP3* deficiency.

This study involved the recruitment of nine Chinese patients with *GTPBP3* defects. We conducted in-depth analyses of clinical information and carried out cytological function experiments to confirm the pathogenicity of the novel variants and broaden the genetic spectrum of *GTPBP3*. These findings will contribute to a deeper understanding of the intricate structure and function of the GTPBP3 protein, while also offering valuable insights for clinical diagnosis.

## Results

### Clinical presentations

**Fig. 1:**
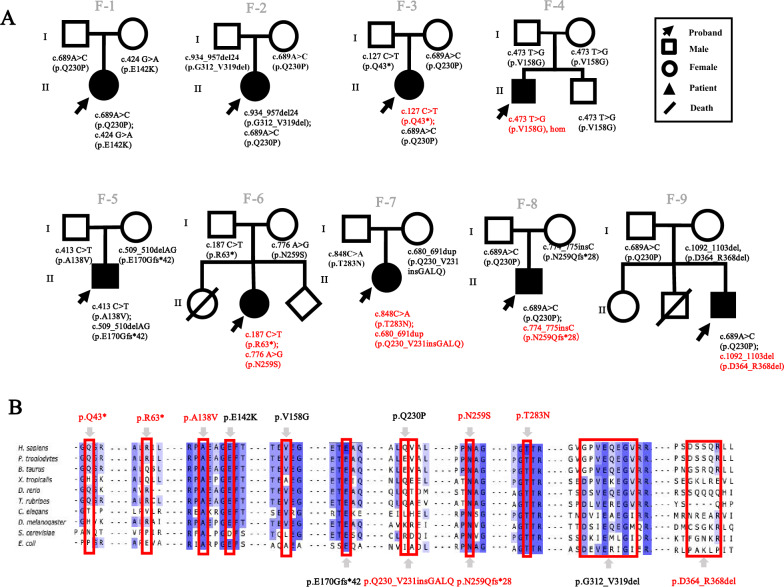
Pedigree diagram of patients and amino acid conservative analysis. **a** Pedigree map of family 1–9. The black arrow indicates the proband, the box indicates the male, the circle indicates the female, the black solid shape indicates the patient, the black slash indicates the death of unknown cause, and the red font indicates the newly discovered variants of *GTPBP3.* **b** The amino acid conservation analysis of variants across species

Patient 1(P1, F-1 in Fig. 1A) is a female who was hospitalized at the age of 1-year-9-month-old due to experiencing fever and seizures three times in a single day. Upon physical examination, she exhibited developmental delay and left ankle clonus. Laboratory tests revealed elevated levels of lactate in the blood (16 mM). Brain MRI indicated abnormalities in the bilateral dorsal thalamus, cerebellar dentate nucleus, and superior cerebellar peduncle. Echocardiography (ECG) revealed left ventricle enlargement and left ventricular wall thickening, which indicates hypertrophic cardiomyopathy. Genetic analysis revealed a *GTPBP3* compound heterozygous mutation: c.689A > C inherited from the father and c.424G > A inherited from the mother.

Patient 2 (P2, F-2 in Fig. 1A) is a female born through full-term natural delivery. She exhibited delayed motor milestones and could not stand on her own at 9 months of age. At 1-year-1-month old, she was hospitalized due to vomiting for 4 consecutive days. Physical examination revealed hypotonia and hyperreflexia in the knee tendons. Blood lactate levels were elevated to 8.58 mM. Brain MRI showed abnormal signals in the bilateral thalamus and peduncle. Electroencephalogram (EEG) indicated diffuse delta waves as the predominant slow wave pattern, and echocardiography revealed decreased left ventricular function (ejection fraction (EF) = 54%) and mild regurgitation of the second, tricuspid, and pulmonary valves. Genetic testing confirmed compound heterozygous variants in *GTPBP3*: c.934_957del inherited from her father and c.689A > C inherited from her mother.

Patient 3 (P3, F-3 in Fig. 1A) experienced a seizure with coma lasting 2 h at the age of 3 years and 8 months. Her condition rapidly deteriorated, presenting with unconsciousness, abnormal breathing patterns, decreased oxygen saturation, and impaired liver function accompanied by uncorrectable metabolic acidosis. Laboratory examination revealed a blood glucose level of 27 mM, blood lactate level of 16 mM, blood ammonia level of 94 μM, alanine aminotransferase of 283.3U/L, aspartate aminotransferase of 716.6 U/L. Brain MRI revealed abnormal signals in the bilateral thalamus, peduncle, and bilateral temporoparietal cortex. EEG showed a slowing of basic waves, and ECG revealed that the left atrium and left ventricle were slightly enlarged, and the left ventricular systolic function was normal (EF = 55%, FS = 28%). Genetic examination revealed compound heterozygous variants in *GTPBP3*: c.689A > C (paternally inherited) and c.127C > T (maternally inherited).

Patient 4 (P4, F-4 in Fig. 1A) is a young boy who was admitted to the hospital at the age of 4 years and 8 months due to weakness that had persisted for over 2 years. He experienced fatigue easily, had poor endurance. Upon physical examination, it was noted that his growth and development were borderline normal, and he had astigmatism. Laboratory tests revealed a high-sensitive troponin-I level of 0.026 ng/mL, NT-proBNP of 894 pg/ml, CK-MB of 21 U/L, and blood lactate level of 9.18 mM. Ultrasonography showed left ventricular enlargement, ventricular wall hypertrophy, and an EF of 38%, consistent with a diagnosis of heart failure with grade III cardiac function. Genetic testing revealed a homozygous variant in *GTPBP3* (c.473 T > G).

Patient 5 (P5, F-5 in Fig. 1A) is a male infant who presented with rapid, shallow breathing and intermittent moaning starting at 20 h after birth. His blood lactate level was significantly elevated at 26 mM, indicating metabolic acidosis along with respiratory acidosis. Additionally, neonatal disease screening using LC/MS revealed a marked increase in alanine. Genetic examination revealed *GTPBP3* compound heterozygous mutation: c.413C > T inherited from her father and c.509_510del inherited from her mother.

Patient 6 (P6, F-6 in Fig. 1A) is a female child born to healthy, unrelated parents. Her sibling passed away at 7 months due to a “brain disease”. Shortly after birth, developmental delays were noted in the child, presenting with symptoms of unsteady running, speech disorder, and limited comprehension. By the age of 2, she experienced sporadic seizures associated with colds and fever once or twice a year. Physical examination revealed increased muscle tone in the limbs, left eye esotropia, and restricted external rotation. Elevated blood lactic acid levels at 6.94 mmol/L were observed, along with abnormal signals in the bilateral thalamus and left cortex on brain MRI. Additionally, the ECG displayed two generalized spikes and slow spikes during sleep. Cardiac ultrasound indicated mild regurgitation in the tricuspid valve, main arteries, and pulmonary arteries. Genetic testing identified a compound heterozygous mutation in *GTPBP3*: c.187C > T (paternally inherited) and c.776A > G (maternally inherited).

Patient 7 (P7, F-7 in Fig. 1A) is a 3-year-old girl who was hospitalized due to multiple seizures. Genetic examination revealed *GTPBP3* compound heterozygous mutation c.848C > A (paternally inherited) and c.680_691dup (maternally inherited).

Patient 8 (P8, F-8 in Fig. 1A), a male, was hospitalized at the age of 2 due to fever and seizures. He exhibited delayed motor development, weak muscle tone, hypotonia, and fatigue. Elevated blood lactate levels were recorded at 8.3 mM. Brain MRI displayed abnormal hyperintensity signals in the basal ganglia. Genetic examination revealed *GTPBP3* compound heterozygous mutation c.689A > C (paternally inherited) and c.774_775insC (maternally inherited).

Patient 9 (P9, F-9 in Fig. 1A) is the third child born to unrelated parents. His sister is normal, and his brother passed away at the age of 1 year and two months of unknown etiology. He presented with language development delay and hypotonia of both lower limbs. Brain MRI revealed suspicious white matter abnormality. Genetic examination revealed *GTPBP3* compound heterozygous mutation: c.689A > C (paternally inherited) and c.1092_1103del (maternally inherited).

Detailed clinical presentations and other examination results are summarized in Table 1.

**Table 1:** Clinical features of 9 patients with *GTPBP3* deficiency

Patient	P1	P2	P3	P4	P5	P6	P7	P8	P9
Gender	Female	Female	Female	Male	Male	Female	Female	Male	Male
Age of onset	1.8 years	1.1 years	3.7 years	4.7 years	At bitrh (20 h)	7 months	3 years	2 years	1 years
Variant	c.689A > C; c.424G > A	c.689A > C; c.934_957del	c.689A > C; c.127C > T	c.473 T > G, hom	c.413C > T; c.509_510del	c.187C > T; c.776A > G	c.680_691dup; c.848C > A	c.774_775insC; c.689A > C	c.689A > C; c.1092_1103del
Clinical diagnosis	LS	LS	LS	MD	MD	LS	LS	MD	MD
Clinical features	developmental delay; Seizure; fatigue	developmental delay; Hypotonia; Knee tendon hyperreflexes	Seizure; fatigue	developmental critical state; fatigue; exercise intolerance	rapid shallow breathing followed by intermittent moaning	developmental delay; Epilepsy; Hypertonia; Knee tendon hyperreflexes	seizure	developmental delay; Seizure; fatigue	Developmental delay; Hypotonia
Echocardiography (EF≧50%; 25%≦FS≦45%)	Cardiac hypertrophy (EF = 72%; FS = 40%)	Low left ventricular function (EF = 54%; FS = 27%)	Slightly larger atria and ventricles, low left ventricular function (EF = 55%; FS = 28%)	Ventricular hypertrophy, slightly reduced left ventricular systolic function, mitral regurgitation (EF = 38–60%; FS = 28.5–45%)	NA	Mild regurgitation of tricuspid valve and main and pulmonary valve	NA	NA	NA
Brain MRI	Bilateral thalamus, cerebellar dentate nucleus and local subcortical brain abnormal signals	Abnormal signals in the bilateral thalamus and peduncle	Bilateral high signal in thalamus, midbrain and peduncle	No obvious change	NA	Multiple asymmetric signal foci in the bilateral thalamus, brainstem and medulla oblongata	NA	Basal ganglia lesion	Suspected white matter abnormality
Plasma lactate level (mM)	16	8.58	16	9.18	NA	5.92	NA	8.3	NA
Others	NA	Abnormal electroencephalogram	Abnormal electroencephalogram	NA	NA	Abnormal electroencephalogram	NA	NA	NA

*LS, Leigh syndrome; MD, mitochondrial disease; NA, not available

### Pathogenicity prediction of variants

Cross-species amino acid conservative analysis (Fig. 1B) was carried out among different variants. Except for c.689A > C (p.Q230P), other residues are highly conserved during evolution. The summary of pathogenicity analysis for genetic variants is presented in Table 2. gnomAD (http://gnomad.broadinstitute.org) was an allele frequency annotations database [18]. The variants were either absent or the frequency was extremely low in the population. SIFT (http://sift-dna.org) and PolyPhen-2 (http://genetics.bwh.harvard.eduy/pph2) were the typical pathogenicity prediction tools for non-synonymous single nucleotide substitution [19, 20]. MutationTaster (https://www.mutationtaster.org) works on the DNA level, also suitable for indels. The score of c.413C > T, c.424G > A, c.473 T > G, c.776A > G, and c.848C > A in SIFT all greater than 0.05. And the score of c.413C > T, c.424G > A, c.473 T > G, c.776A > G and c.848C > A in PolyPhen-2 all greater than 0.909. Almost variants are predicted to be disease-causing in MutationTaster. Moreover, MUpro (http://mupro.proteomics.ics.uci.edu/) was used to predict the change in protein stability [21]. It seems that c.413C > T, c.424G > A, c.473 T > G, c.689A > C, c.776A > G, and c.848C > A were more likely to lead to decreased protein stability.

**Table 2:** The annotations of variants

Variant (NM_032620.4)	Exon	Amino acid change	gnomAD	SIFT	Polyphen-2	MutationTaster	MUpro
c.127C > T	2	p.Q43*	NA	NA	NA	D	NA
c.187C > T	2	p.R63*	NA	NA	NA	D	NA
c.413C > T	4	p.A138V	< 1‰	0.01	1.000	D	Decreased
c.424G > A	4	p.E142K	NA	0.00	1.000	D	Decreased
c.473 T > G	4	p.V158G	NA	0.05	0.999	D	Decreased
c.509_510del	4	p.E170Gfs*42	< 1‰	NA	NA	D	NA
c.680_691dup	6	p.Q230_V231insGALQ	NA	NA	NA	P	NA
c.689A > C	6	p.Q230P	< 1‰	0.25	0.091	D	Decreased
c.774_775insC	6	p.N259Qfs*28	< 1‰	NA	NA	D	NA
c.776A > G	6	p.N259S	< 1‰	0.00	1.000	D	Decreased
c.848C > A	7	p.T283N	NA	0.00	0.999	D	Decreased
c.934_957del	7	p.G312_V319del	< 1‰	NA	NA	D	NA
c.1092_1103del	8	p.D364_R368del	NA	NA	NA	D	NA

*SIFT score: 0.0–0.05 means deleterious and 0.05–1.0 means tolerated; PolyPhen-2 score: 0.0–0.446 is Benign, 0.447–0.908 is possibly damaging and 0.909–1.0 for probably damaging; D-prediction disease causing, P-prediction polymorphism

NA: not found

In summary, a total of thirteen variant sites were involved in nine pedigrees, with five have been reported and eight novel variants. While predictive analyses indicate potential defects in all variants, confirmation through additional biological functional verification is required.

### Decreased GTPBP protein levels and impaired mitochondrial function were observed in patient-derived immortalized lymphocytes

**Fig. 2:**
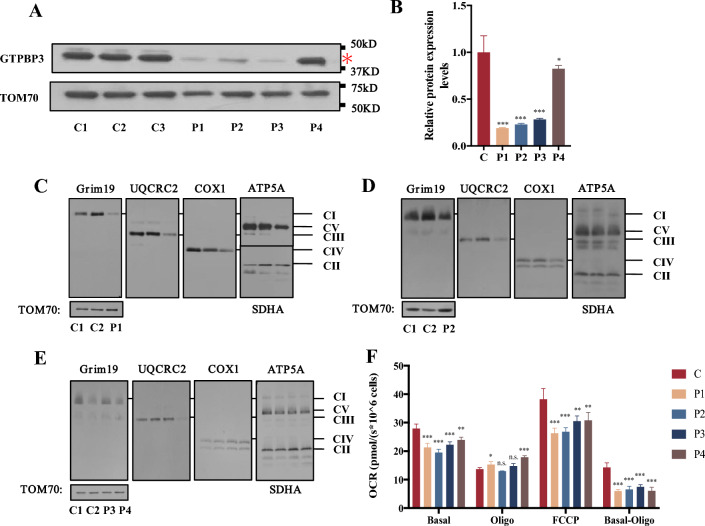
Analysis of GTPBP3 amount in patient-derived immortalized lymphocytes. Analysis of mitochondrial complex content and OCR in patient-derived immortalized lymphocytes. **a** WB analysis of GTPBP3 in immortalized lymphocytes of normal controls and patients. **b** Quantitative analysis of GTPBP3 abundance in immortalized lymphocytes of normal controls and patients (Fig. 2A). TOM70 was used as an internal control (n = 3). The asterisk indicates the target strip. Data are presented as the means ± SEM (n = 3). *p < 0.05, **p < 0.01, ***p < 0.001. ****p < 0.0001. **c-f** Abundance of OXPHOS complexes in immortalized lymphocytes of normal controls and patients. TOM70 was used as a loading control. **g** Oxygen consumption rate analysis in immortalized lymphocytes of normal controls and patients. Basal indicates basal respiration, Basal-Oligo indicates ATP-link OCR, FCCP indicates maximal respiration. The absolute OCR was normalized against the cell number (n = 4). Data are presented as the means ± SEM (n = 3). *p < 0.05, **p < 0.01, ***p < 0.001. ****p < 0.0001

Four patients (P1-P4) and three age-matched healthy children as controls were included in the immortalized lymphocyte experiments. Initial validation of the immortalized lymphocytes was conducted through Sanger sequencing, confirming consistency with the previous genetic examination (Supplementary Fig. 1A). Analysis comparing the steady-state GTPBP3 protein levels in patients P1-P4 with the normal control group revealed significant decreases of 82.8% (*p* < 0.001), 79.1% (*p* < 0.001), 74.4% (*p* < 0.001), and 25.5% (*p* = 0.036) respectively (Fig. 2A, B). These findings highlight the substantial reduction in GTPBP3 protein levels in patient-derived lymphocytes.

As previously mentioned, GTPBP3 is a highly conserved mt-tRNA modifying enzyme essential for the mitochondrial protein translation process [7]. To further elucidate its impact on mitochondrial function, BN-PAGE is a common technique optimized for the analysis of the five complexes (CI–CV) of OXPHOS (Fig. 2C–E) [22, 23]. Compared with the control, the content of CI, CIII, CIV, and CV was decreased in P1 and P2 (Fig. 2C, D). Additionally, in P4, the content of complex CIII was decreased, while no significant differences were observed in the abundance of mitochondrial complexes in P3 (Fig. 2E). To further detect the mitochondrial respiratory capacity, the oxygen consumption levels in lymphocytes were measured [24, 25]. The basal respiration rate was measured under normal conditions. Oligomycin was used to inhibit ATP synthase, allowing for the calculation of the corresponding OXPHOS-related oxygen consumption rate (OCR). FCCP was used to disrupt the proton gradient and mitochondrial membrane potential, stimulating cells to reach their maximum respiration potential [26]. As a result, basal OCR of P1was decreased by 23.7% (*p* < 0.001), P2 decreased by 30.2% (*p* < 0.001), P3 decreased by 20.4% (*p* < 0.001) and P4 decreased by 14.4% (*p* = 0.0012). The OCR of oxidative phosphorylation decreased by 57.8% (*p* < 0.001) in P1, 54.0% (*p* < 0.001) in P2, 47.4% (*p* < 0.001) in P3, and 57.4% (*p* < 0.001) in P4. The maximum respiration potential of P1 was decreased by 31.3% (*p* < 0.001), P2 was decreased by 29.9% (*p* < 0.001), P3 was decreased by 20.1% (*p* = 0.0018) and P4 was decreased by 19.4% (*p* = 0.0024) (Fig. 2F). In summary, the OXPHOS function of P1-P4 was impaired to varying degrees.

### Re-expression of wild-type vectors rescues the deficit in GTPBP3 expression level and OXPHOS complexes

**Fig. 3:**
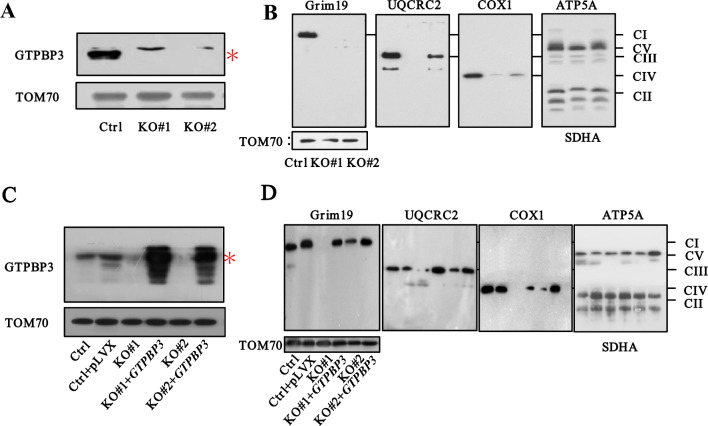
Identification of HEK293T *GTPBP3* KO and re-expression cell model. **a** The abundance of GTPBP3 in HEK293T of KO and control (Ctrl) cells. TOM70 was used as a loading control. **b** The BN-PAGE analysis of GTPBP3 in HEK293T of KO and Ctrl. TOM70 was used as a loading control. **c** The abundance of GTPBP3 in HEK293T of KO, KO transfected with *GTPBP3* and Ctrl. TOM70 was used as a loading control. **d** The BN-PAGE analysis of GTPBP3 in HEK293T of KO, transfected with *GTPBP3* and Ctrl. TOM70 was used as a loading control

To further investigate the impact of GTPBP3 on mitochondrial functions, we utilized CRISPR-Cas9 technology to generate a HEK293T *GTPBP3* knockout (KO) cell model, which was then rescued by re-expressing wild-type GTPBP3. Western blot analysis confirmed the reduced level of GTPBP3 protein in the KO cell model (Fig. 3A). Additionally, blue native polyacrylamide gel electrophoresis (BN-PAGE) revealed significant decreases in Complexes I, III, IV, and V (Fig. 3B), consistent with findings in patient-derived lymphocyte models. Subsequent analysis of the re-expressed cells through WB and BN-PAGE demonstrated partial recovery of the observed defects (Fig. 3C, D).

### Protein abundance decreased in *GTPBP3* site-directed mutagenesis cell model

According to instructions of the Standard and guidelines for the interpretation of sequence (2015) published by the American Society for Medical Genetics and Genomics (ACMG) [27], nonsense mutations, frameshifts, ± 1 or 2 canonical splice sites, initiation codons, and large deletions, all have pathogenic very strong evidence, combined with extremely low population frequency, they can be distributed to Likely pathogenic (LP) at least. Cytological function experiments can provide strong evidence for pathogenicity analysis, which was a milestone significance for VUS variants [28, 29]. Re-expressing *GTPBP3* carrying mutant vectors of c.127C > T, c.187C > T, c.473 T > G, c.776A > G, c.848C > A and mutation hot spot (c.689A > C) vectors in *GTPBP3* KO cells, mutations were identified by Sanger sequencing (Supplementary Fig. 1B).

**Fig. 4:**
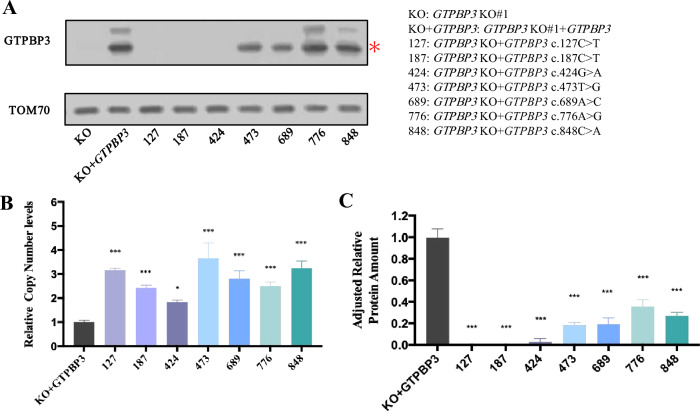
GTPBP3 protein expression level analysis of HEK293T cell lines carrying different vatiants. **a** The abundance of GTPBP3 in HEK293T with KO, KO transfected with *GTPBP3*, KO transfected with *GTPBP3* carrying different variants (sites can be seen in the panel). TOM70 was used as a loading control. **b** Relative plasmids copy number levels in HEK293T with KO transfected with *GTPBP3* and KO transfected with *GTPBP3* carrying different variants. β-Actin was used as an internal control (n = 3). **c** Quantitative results of relative abundance were corrected by relative plasmid copy number levels. Data are presented as the means ± SEM (n = 3). *p < 0.05, **p < 0.01, ***p < 0.001. ****p < 0.0001

To eliminate the interference of wild-type GTPBP3 protein, the *GTPBP3* vectors carrying different mutations were transfected into KO cell lines. As shown in Fig. 4A, when compared with KO + *GTPBP3* cell line, KO + c.127C > T decreased by 97.8% (*p* < 0.001), KO + c.187C > T decreased by 99.6% (*p* < 0.001), KO + c.424G > A decreased by 94.8% (*p* < 0.001), KO + c.473 T > G decreased by 32.9% (*p* = 0.0064) and decreased by 45.7% (*p* < 0.001) in KO + c.689A > C. No significant decrease was found in KO + c.776A > G and KO + c.848C > A. It needs to be considered that there are differences in vector copy number among cell lines [30, 31]. We designed primers targeting to the 3' end of the CDS and PGK promoter which was a conserved region on vectors to assess the relative level of vectors. As shown in Fig. 4B. The vector levels of each cell lines were compared with KO + *GTPBP3* cell line, KO + c.127C > T was 3.2 times (*p* < 0.001), KO + c.187C > T was 2.4 times (*p* < 0.001), KO + c.424G > A was 1.8 times (*p* = 0.0118), KO + c.473 T > G was 3.7 times (*p* < 0.001), and KO + c.689A > C was 2.8 times (*p* < 0.001), KO + c.776A > G was 2.5 times (*p* < 0.001), KO + c.848C > A was 3.2 times (*p* < 0.001), KO + c.1384C > G was 4.8 times (*p* < 0.001). Once the relative efficiency level further corrected the protein content, the masked differences of KO + c.776A > G and KO + c.848C > A can be uncovered (Fig. 4C).

### Analysis of genetic variants spectrum and phenotype-genotype correlation of *GTPBP3*

**Fig. 5:**
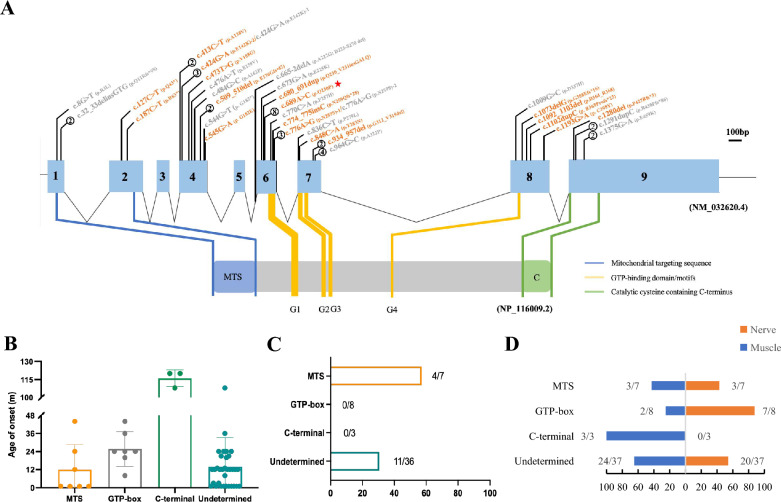
Mutations spectrum of *GTPBP3* and phenotype-genotype correlation analysis. **a** Updated mutation map of *GTPBP3.* The orange font represents the mutations found in Chinese population, and the gray font represents the mutations reported in other populations; The number in the circle indicates the frequency of occurrence, and the unmarked frequency is 1; The asterisk indicates the hot spots of the population; The blue box indicates the exon, and the number in the blue box indicates the number of exon; Purple, yellow, and green boxes represent different functional domains of the corresponding proteins, respectively. **b** The age of onset distribution of patients deficit in *GTPBP3*. **c** The percentage of death outcomes of patients deficit in *GTPBP3*. **d** The distribution of patients presented with nerve and/or muscle systems

The initial report by Robert Kopajtich et al. in 2014 documented 11 cases of *GTPBP3* mutations [11]. To date, in total of 21 cases with 23 distinct variants of *GTPBP3* have been reported. By incorporating these reported variants with the 8 novel variants identified in our study, the genetic spectrum of the *GTPBP3* gene was expanded (Fig. 5A). Notably, variants identified in the Chinese population are marked in yellow (Fig. 5A), we noted that c.689A > G is most common in the Chinese population. Moreover, our analysis revealed that the variant sites are predominantly concentrated in exon 4 and exon 6, with c.689A > C showing high frequencies of 8/51, indicating it as a hot spot mutation site within this population.

Based on the protein’s functional domains, the mutations can be roughly categorized into four regions, which are mitochondrial targeting sequence (MTS, M), GTP-box (G), C-terminal (C), and other undetermined (U) regions [11]. Combined with previous cases, the characteristics were summarized as follows (Fig. 5B–D). Firstly, the onset age for all cases was under 10 years old, with a trend to earlier onset in the M/U region (Fig. 5B). Secondly, the clinical outcomes of patients in the M/U region more servere, with a higher proportion mortality ratio (Fig. 5C). In terms of muscle involvement, individuals in the M/G/U region were usually affected by dual involvement. Those in the C region tended to exhibit muscle involvement, along with energy deficiency along with energy deficiency symptoms like fatigue and mild myocardial hypertrophy (limited by the small number of case samples) (Fig. 5D). Overall, it appears that symptoms in the C region were relatively mild, may due to the smaller sample size. Lastly, all patients exhibited hyperlactatemia, and the majority experienced developmental delay along with muscle and/or nerve involvement (such as cardiac hypertrophy, seizures, hypermyotonia, or hypotonia) and abnormal brain MRI. They may also present with dyspnea, feeding difficulties, short stature, and occasionally visual impairment, as well as cardiac abnormalities. These cardiac abnormalities can include conduction and heart valve issues. Notably, valvular insufficiency was initially linked to *GTPBP3* deficiency in our study.

## Discussion

GTPBP3 is a catalytic enzyme involved in the synthesis of τm^5^(s^2^) U in mitochondria and has been associated with mitochondrial diseases. Since Robert Kopajtich first described the phenotypes associated with 11 cases of *GTPBP3* deficiency [11]. At present, there are only several sporadic cases. However, restricted by the small number of cases, the disease-related phenotypes are incomplete, and the mutation spectrum still needs to improve. The correlation between genotype and phenotype remains to be further studied. According to previous studies, changes in oxidative phosphorylation complex enzyme activity of skeletal muscle and fibroblasts, several mitochondrial subunits protein levels as well as mitochondrial oxygen consumption rate under different culture conditions in patient-derived fibroblasts have been presented [11]. However, there are still few studies on patient-derived cell models, and the studies are only for individuals, and the subjects are inconsistent. We constructed patient-derived immortalized lymphocyte cell lines and performed BN-PAGE to preserve their native structure of OXPHOS complexes and subsequently oxygen consumption rates detection. Our results agree with the conclusion that *GTPBP3* deficiency led to mitochondrial dysfunction. However, the new phenotypes including heart valve involvement and strabismus were first proposed in this study.

The level of residual steady-state GTPBP3 protein seems to be correlated with the degree of mitochondrial impairment, except for P3, which may be due to the following hypotheses: (1) The produced mitochondrial complex protein is useless in this patient, so the protein level does not change significantly, but the activity of complex enzyme is severely impaired [25]; (2) The disadvantage of immortalized lymphocytes is that the phenotype is not obvious [32, 33]; (3) Cells were cultured in a high-nutrient environment, and excessive reliance on glycolysis can compensate for the deficiency. In other words, stress culture can make the difference obviously [33, 34]. In the results of GTPBP3 protein level detection on site-directed mutagenesis cell models, c.776A > G and c.848C > A were inconsistent with the predicted results. The possible reasons were as follows: (1) The mutations did not affect the protein level but affected the enzyme catalytic activity [25]; (2) Different transfection and replication efficiency of plasmids would affect the protein expression level 35, 36. The results showed that the plasmids expression level in all site-mutant cell lines was higher than control. As a result, the pathogenicity of c.776A > G and c.848C > A cannot be ruled out.

The reduction of protein level may be due to the decrease in protein production and/or the acceleration of protein degradation [37–39]. Previous studies have shown that the GTPBP3 protein is extensively modified by ubiquitination and degraded through the proteasome pathway 7. To avoid rapid protein degradation, we first treated the cells with MG-132 for 6 h, then inhibited cell protein synthesis by CHX treatment, the degree of degradation of target protein can be observed within 24h [38, 40–42] (Supplementary Fig. 2A). It was found that compared with the control group, c.127C > T and c.187C > T significantly decreased after 6 h of treatment, suggesting that c.127C > T and c.187C > T accelerated the degradation of GTPBP3 protein and reduced the stability of the protein (Supplementary Fig. 2B).

In summary, we enrolled 9 individuals with *GTPBP3* deficiency. We identified 8 novel variants, which were c.127C > T, c.187C > T, c.473 T > G, c.680_691dup, c.774_775insC, c.776A > G, c.848C > A and c.1092_1103del, respectively. 7 mutations were screened to construct site-directed mutagenesis cell models and cytological function experiments were performed. It was confirmed that c.127C > T, c.187C > T, c.424G > A, c.473 T > G, and c.689A > C were pathogenic mutations. *GTPBP3* mutation spectrum was expanded, and it was found that mutations in the Chinese population were mostly concentrated in exon 4 and exon 6, and c.689A > C and c.424G > A were hot spots in the population. This study highlights the important role of mt-tRNA modification defects in mitochondrial diseases and provides a reference for the diagnosis of diseases related to *GTPBP3* deficiency, as well as subsequent prenatal diagnosis and genetic counseling.

## Methods

### Study participant

Patients were born from 9 non-consanguineous families. Patients 5 and 6 underwent evaluation at Xiangya Hospital, Central South University, while other patients were recruited and assessed at Peking University First Hospital. Approval for this study was obtained from the Ethics Committees of both Peking University First Hospital (Ethics approval number: 2017–217) and Xiangya Hospital (Ethics approval number: 201605585). Moreover, informed consent was obtained from all participants or guardians.

### Genetic analysis

DNA was extracted from peripheral blood samples of probands and their parents. Whole exome sequencing (WES) and mitochondrial genome sequencing were performed using the HiSeq 2000 sequencer (Illumina, USA). Sanger sequencing was then carried out as a follow-up to validate the identified mutations [43, 44]. The specific primers used are detailed in Supplementary Table 1.

### Immortalized lymphocytes construction

As previously outlined [45, 46], mononuclear cells were isolated from the peripheral blood using the lymphocyte separation medium (Solarbio, China). These isolated cells were continuously stimulated by Epstein-Barr virus (EBV). Furthermore, 0.5 mg/mL phytohemagglutinin (Sigma-Aldrich, USA) and 1 mg/mL cyclosporin A (Sigma–Aldrich) were also added to the culture medium.

### Plasmids construction and transfection

As previously described [46, 47], Knockout (KO) plasmids were constructed using the CRISPR/Cas9 technology, and gRNAs were annealed to duplexes and inserted into pX330 vector. As for overexpression (OE) plasmid, *GTPBP3* was synthesized by Phanta Max Super-Fidelity DNA Polymerase (Vazyme, China) and cloned into lentiviral pLVX vector by ClonExpression® II One Step Cloning Kit (Vazyme), and site-specific mutant vectors were obtained from Tsingke (Tsingke Biotechnology, China). All constructions were confirmed by Sanger sequencing. Transfection was performed with Lipofectamine 3000 reagent (Invitrogen, USA) according to the manufacturer’s instructions. KO cell lines were selected by limiting dilution, and OE cell lines and site-mutant cell lines were generated by infection of the cells with lentiviral particles and puromycin (Sangon Biotech, China) selection.

### Cell culture

Immortalized lymphocytes from patients were cultured in RPMI 1640 medium (Thermo Fisher Scientific) supplemented with 10% fetal bovine serum (GIBCO, USA) and 1% penicillin/streptomycin, as well as 50 mg/mL uridine (Sigma-Aldrich).

HEK293T was a gift from Dr. Haihua Gu (Wenzhou Medical University). HEK293T and other cell models generated from HEK293T were cultured in Dulbecco’s modified Eagle medium (DMEM, Thermo Fisher Scientific, USA) containing 12% calf serum (Sigma-Aldrich) and 1% penicillin/streptomycin. 2 μg/mL puromycin (Sangon, China) was extremely added to cell models generated from HEK293T. All cells were cultured with 5% CO_2_ at 37 °C in an incubator.

### Quantitative real-time PCR (qPCR) detection

RNA was extracted according to the TRIzol Reagent protocol (Thermo Fisher Scientific) 48. Complementary DNA (cDNA) was synthesized by PrimeScript RT reagent Kit (Takara Biotechnology, Japan). For quantification transcripts expression level, qPCR was performed with 2xChamQ SYBR qPCR Master Mix (Vazyme, China). Primers are provided in Supplementary Table 1.

### Immunoblotting

For sodium dodecyl sulfate–polyacrylamide gel electrophoresis (SDS-PAGE), a total protein isolated from whole cell using RIPA lysis buffer (Cell Signaling Technology, USA) with 1 mM phenylmethylsulfonyl fluoride (PMSF, Sangon Biotech, China).

For Blue native polyacrylamide gel electrophoresis (BN-PAGE), as previously described 22, mitochondrial membrane protein was extracted from a whole cell using 2% Triton-X100 (Sigma-Aldrich) and subsequently separated by a 3.5%−16% gradient gel. Proteins were electroblotted onto 0.22 um PVDF membranes (Bio-rad, USA) and blocked with 5% milk powder solution, incubated with primary and secondary horseradish peroxidase-conjugated antibodies (Supplementary Table 2). Signals detected with clarity ECL western blotting (WB) substrate (Bio-Rad).

### Mitochondrial respiration measurement

The detection of oxygen consumption rate (OCR) was conducted as described before [44, 49]. Concisely, about 5 × 10^6^ immortalized lymphocytes were harvested and added to the chamber of Oxygraph-2 k (Oroboros, Austria). The respiration was recorded under normal conditions and with subsequent injection with inhibitors, including oligomycin (0.1 mM, Sigma–Aldrich) and carbonyl cyanide 4-(trifluoromethoxy) phenylhydrazone (FCCP, 0.1 mM, Sigma–Aldrich).

### Cycloheximide (CHX) chase assay

Cells were seeded on 24-well cell culture plates (10,000 cells per well). Once the confluence reached 90%, cells were pretreated with a complete medium containing 10uM MG132 for 6 h. After washing cells twice with PBS, the culture medium was converted to a complete medium containing 20uM cycloheximide (CHX). Samples were harvested at 0, 3, 6, 12, and 24 h after CHX treatment for SDS-PAGE detection.

### Statistical analysis

All quantitative data were performed for three or more times, independently. Statistical analysis and graphs were plotted using Prism 8.4.0. The results were shown with mean ± SD. When the data conforms to a normal distribution, independent double-tailed student t was used. If not, the Mann–Whitney U test is used. Three or more groups of data were compared using one-way analysis of variance (ANOVA), *p* < 0.05 indicates statistical significance, *p < 0.05, **p < 0.01, ***p < 0.001.

## Supplementary Information

Additional file 1: Fig. S1 Sanger Sequencing of patients and *GTPBP3* mutant plasmids. Sanger sequencing of 4 patient-derived immortalized lymphocytes. Sanger sequencing of 7 constructed plasmids inserted with *GTPBP3* carrying different variants. Fig. S2 Stability analysis of GTPBP3 protein expression level analysis of HEK293T cell lines carrying different variants. The WB of *GTPBP3*-KO carrying different variants plasmidscell lines treated with CHX for 0-24 h. TOM70 was used as a loading control. An asterisk indicates the target strip.The relative abundance of Figure S2A were corrected by relative plasmid copy number levels. Data are presented as the means ± SEM. *p < 0.05, **p < 0.01, ***p < 0.001, ****p < 0.0001.

Additional file 2: Table S1. The sequence of primers and gRNAs. Table S2. The antibodies for immunoblotting.

## Data Availability

The datasets generated and/or analyzed during the current study are not publicly accessible due to the confidentiality and ethical considerations associated with patient data. However, these datasets can be obtained from the corresponding author upon request.
