# Supplementary figures and images for: Expanding the phenotypic and genetic spectrum of GTPBP3 deficiency: findings from nine Chinese pedigrees
Source: Orphanet J Rare Dis. 2024 Dec 24;19:488. doi: 10.1186/s13023-024-03469-3 (PMC11668094; doi:10.1186/s13023-024-03469-3)

Supplementary Figure 1

A

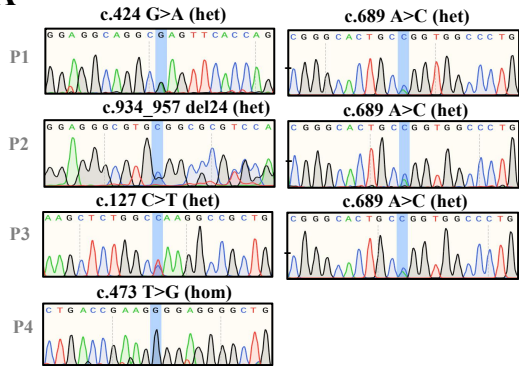

B

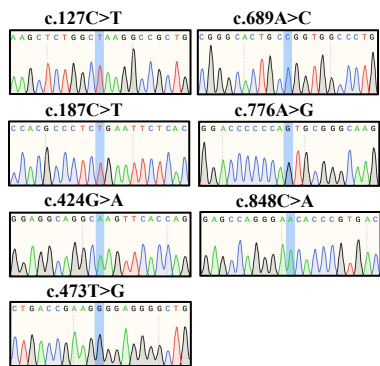

Supplementary Figure 2

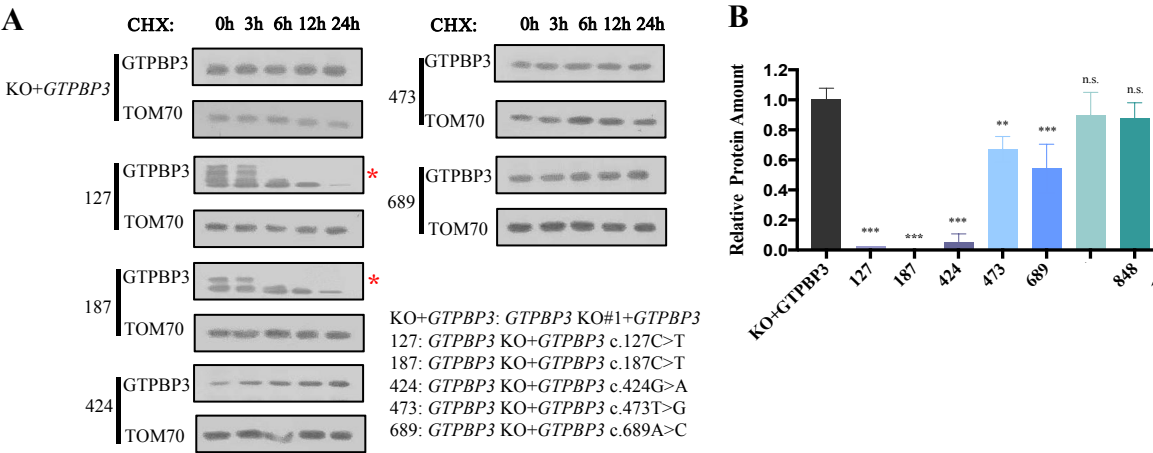

Supplement: Supplementary file 1 — Additional file 1: Fig. S1 Sanger Sequencing of patients and GTPBP3 mutant plasmids. Sanger sequencing of 4 patient-derived immortalized lymphocytes. Sanger sequencing of 7 constructed plasmids inserted with GTPBP3 carrying different variants. Fig. S2 Stability analysis of GTPBP3 protein expression level analysis of HEK293T cell lines carrying different variants. The WB of GTPBP3-KO carrying different variants plasmidscell lines treated with CHX for 0-24 h. TOM70 was used as a loading control. An asterisk indicates the target strip.The relative abundance of Figure S2A were corrected by relative plasmid copy number levels. Data are presented as the means ± SEM. *p < 0.05, **p < 0.01, ***p < 0.001, ****p < 0.0001. [file 13023_2024_3469_MOESM1_ESM.pdf]
