# Supplementary material for: Expanding the phenotypic and genetic spectrum of GTPBP3 deficiency: findings from nine Chinese pedigrees
Source: Orphanet J Rare Dis. 2024 Dec 24;19:488. doi: 10.1186/s13023-024-03469-3 (PMC11668094; doi:10.1186/s13023-024-03469-3)
Supplement: Supplementary file 2 — Additional file 2: Table S1. The sequence of primers and gRNAs. Table S2. The antibodies for immunoblotting. [file 13023_2024_3469_MOESM2_ESM.docx]

**Supplementary Table 1.** The sequence of primers and gRNAs.

| **Primer name** | **Forward primer (5' to 3')** | **Reverse primer (5' to 3')** |
| --- | --- | --- |
| Copy number | GGACTTCTGTGTGGGCAAGTG | GCATGCTCCAGACTGCCTTG |
| β-Actin | GACCTGTACGCCAACACAGT | AGTACTTGCGCTCAGGAGGA |
| sgRNA-1 | CACCGACCATCTTCGCGCTAAGCTC | AAACGAGCTTAGCGCGAAGATGGTC |
| sgRNA-2 | CACCGCCAGAGCTTAGCGCGAAGA | AAACTCTTCGCGCTAAGCTCTGGC |
| *GTPBP3*-OE-amplify | GAGCTCAAGCTTCGAATTCATGTGGCGGGGGCTTTGGACC | ACCGTCGACTGCAGAATTTCACTTGCCCACACAGAAGT |
| c.127-ss | TTCCGTGCCTCAATCGTTCA | GGACCAAGCATCTCACCCAA |
| c.424/473-ss | GTGCTGTCCTCCTGTCACCT | GAGAATGGACAAATGGGGGACT |
| c.689/c.934_957-ss | GTGTGGGAAGGTGGGTTTCT | TGAGATCCATGCTTGATAGGGC |
| Plasmid-ss | AGCTGGACGGAGAGCTGG | GAAAAGCGCCTCCCCTACC |

**Supplementary Table 2.** The antibodies for immunoblotting.

| **Antibodies** | **Source** | **Item number** |
| --- | --- | --- |
| anti-Grim19 | Abcam | ab110240 |
| anti-SDHA | Abcam | ab14715 |
| anti-UQCRC2 | Abcam | ab14745 |
| anti-MT-CYB | Abcam | ab219823 |
| anti-MT-CO1 | Abcam | ab14705 |
| anti-MT-CO2 | Abcam | ab110258 |
| anti-ATP5A | Abcam | ab14748 |
| anti-β-actin | Santa Cruz Biotechnology | sc-47778 |
| anti-TOM70 | ProteinTech | 14528 |
| anti-mouse IgG, HRP-linked antibody | Cell Signaling Technology | 7076 |
| anti-rabbit IgG, HRP-linked antibody | Cell Signaling Technology | 7074 |
